# Effect of harvest time on the compositional changes in essential oils, cannabinoids, and waxes of hemp (*Cannabis sativa* L.)

**DOI:** 10.1098/rsos.211699

**Published:** 2022-06-15

**Authors:** Pakin Noppawan, Camille Bainier, Alexandra Lanot, Simon McQueen-Mason, Nontipa Supanchaiyamat, Thomas M. Attard, Andrew J. Hunt

**Affiliations:** ^1^ Materials Chemistry Research Center, Department of Chemistry and Center of Excellence for Innovation in Chemistry, Faculty of Science, Khon Kaen University, Khon Kaen 40002, Thailand; ^2^ Department of Chemistry, Green Chemistry Centre of Excellence, University of York, Wentworth Way, York YO10 5DD, UK; ^3^ Department of Biology, Centre for Novel Agricultural Products, University of York, Wentworth Way, York YO10 5DD, UK; ^4^ RX Extraction Ltd., Unit 10, Rowen Trade Estate, Neville Road, Bradford BD4 8TQ, UK

**Keywords:** hemp, composition, cannabidiol, waxes, tetrahydrocannabinol, extraction

## Abstract

Demand for cannabinoid is growing, with the global market expected to reach $9.69 billion by 2025. Understanding how chemical composition changes in hemp at different harvest times is crucial to maximizing this industrial crop value. Important compositional changes in three different compound classes (essential oils, cannabinoids, and lipids) from inflorescences (tops), leaves, and stems of hemp (*Cannabis sativa* L., Finola variety) at different harvesting stages have been investigated. Over 85% of the total extracts from the tops were cannabinoids, while leaves demonstrated the greatest quantities of wax ester and sterols. Essential oil and cannabinoid increased in tops until full flowering (third harvest), reaching 2030 µg g^−1^ and 39 475 µg g^−1^, respectively. Cannabinoids decreased at seed maturity (final harvest) to 26 969 µg g^−1^. This demonstrates the importance of early harvesting to maximize cannabidiol (CBD), which is highly sought after for its therapeutic and pharmacological properties. A total of 21 161 µg g^−1^ of CBD was extracted from the tops at full flowering (third harvest); however, a significant increase (63%) in the banned psychoactive tetrahydrocannabinol (THC) was observed from budding (157 µg g^−1^ of biomass) to the full flowering (9873 µg g^−1^ of biomass). Harvesting the tops after budding is preferable due to the high CBD content and low amounts of THC.

## 1. Introduction

Hemp (*Cannabis sativa* L.) is a C3 plant, which has been cultivated for centuries [1]. Its unique composition has enabled it to be used in a variety of applications [1,2]. Hemp can be traditionally classified into two main types. First, there is fibre hemp, which, as the name implies, is cultivated for the high-quality bast fibres (the outer part of the stem) that have extensive use in the paper-manufacturing industry, automotive industries, textile industries, and for thermal insulation (in place of mineral fibres and plastics) [2,3]. These varieties are harvested quite early, at the flowering period, to prevent the formation of lignin in the stem. Second, there are seed varieties, whereby the hemp is grown for the production of seeds in order to obtain hemp seed oil, a high-quality oil rich in omega-3 content [4,5]. These varieties are harvested much later than the fibre hemp, once the majority of the seeds have reached maturity [3]. Although industrial genotypes of hemp are primarily cultivated for fibre or seed production, the extraction of valuable natural products can offer significant additional financial advantages. Development of natural ingredients for use in food, beverage, nutraceutical, and pharmaceutical products is of ever-growing importance with the increasing demand by consumers for natural products. There are several lipophilic molecules found within hemp threshing residues that have the potential to be used in such products [6].

Knowing the biomass and metabolite yields at various stages of hemp maturation is key for holistic valorization of these resources. This is especially true in the case of the seed varieties, as a significant amount of waste residues, referred to as ‘threshing residues’, are generated during the harvesting of hemp for seed production. Threshing residues consist of the inflorescences (tops), leaves, and stems.

Finola is an early maturing genotype of hemp, which is primarily grown for seed and possesses a high inflorescence yield, but compared with other varieties it exhibits lower amounts of dried biomass (stem and leaves) [7,8]. Importantly, this genotype reaches full flowering after 62 days, which is significantly shorter than other genotypes that reach this stage at 80 days [9]. There have been reports on phytochemical analysis of inflorescences and seeds of the Finola hemp variety [10]. However, limited research has been conducted on the lipid composition of leaves and stems of Finola.

Different parts of hemp, including stems, leaves, inflorescences, and seeds, are the sources of numerous chemicals such as terpenes, carbohydrates, fatty acids, phytochemicals, phenolic compounds, and cannabinoids that have been linked to health-promoting properties [11]. The stem is normally used in textile and paper productions and also used as animal feed due to its cellulose and woody fibres, while the leaf is considered as waste in the fibre industry [11]. The tops or inflorescence are the parts of the plant that contain essential oils, in particular myrcene, which is used in the perfume industry [12]. Hemp leaf and stem residues are normally discarded leading to a loss of potential sources of added-value compounds such as waxes and cannabinoids, which could be used in a variety of applications from nutraceuticals and pharmaceuticals to lubricants, detergents, and cosmetics [13–19]. Cannabinoids are highly sought after for their wide range of pharmacological activities. Cannabinoids are found in all parts of *Cannabis sativa*, but the highest concentration is found in glandular trichomes on the leaf and inflorescence flower surfaces [20]. Particularly, cannabidiol (CBD) is significantly important to a variety of industrial sectors due to the high demand for incorporation into consumer products (cosmetics, food and drink, nutraceuticals) and pharmaceutical products as well as the high market price—CBD distillate sells for $3000 kg CBD^−1^ while CBD isolate sells for $1000 kg CBD^−1^. To valorize this waste residue, it is vital to understand the lipophilic composition as well as its variation over time.

Previous studies have looked into the extraction of essential oils from the female flower inflorescences and leaflets by steam distillation [21,22]. The main compounds found in the hemp essential oil are sesquiterpenes and monoterpenes, including myrcene, *α*-pinene, *β*-pinene, *β*-caryophyllene, *trans*-ocimene, humulene, and terpinolene. There have been limited reports on the use of Soxhlet extractions of essential oils from hemp, but they have been widely carried out on other plants, with the main solvents used being n-hexane [23–27], ethanol [23,25,28–30], petroleum ether [27,31], and methanol [25]. Recently, Ricci *et al.* [32] reported the chemical composition of hemp by comparing the extraction techniques, including Soxhlet extraction. The main essential oils from Soxhlet extraction of inflorescences of hemp by using ethanol as the solvent were caryophyllene (54.78%) and humulene (14.13%) [32].

Limited research has focused on the effect of maturation on the lipophilic composition of leaf and stem threshing residues of hemp seed varieties. Ascrizzi *et al.* [33] studied the essential oil yield and composition of inflorescence (threshing residues) of hemp (Fedora 17 variety) in two cultivation sites (lowland and upland of the Pisa province, Tuscany, central Italy) at two different harvest times (August and September). For both sites, the yields of essential oil extraction were slightly higher in the earlier harvest (August), with the greatest abundance of sesquiterpenes and significant amounts of monoterpene hydrocarbons; however, the oxygenated monoterpenes increased in the late harvest (September) [33]. Likewise, Abdollahi *et al*. [34] reported the effect of different stages of maturation on essential oil yield and composition of four hemp varieties: two monoecious non-native (Fedora 17 and its progeny) and two dioecious native (Fars and Yazd). The study examined three different materials: foliage at the vegetative stage, inflorescences at the flowering stage, and inflorescences at the seed maturing stage. Non-native samples produced the highest oil yield in the vegetative stage, with the highest amount of *E*-caryophyllene. During the development of the plant, the ratio of sesquiterpenes to monoterpenes differed significantly, with content of the latter much lower at the vegetative stage than the flowering stage [34].

The impact of harvest times on lipid composition of hemp seed oil from two cultivars, Futura 75 and Carmagnola, at three ripening stages during August and September has also been investigated [35]. In both varieties, the ratio of polyunsaturated to saturated fatty acid increased during ripening, while total sterol content with the greatest abundance of *β*-sitosterol increased and deceased with ripening in Futura 75 and Carmagnola, respectively. In addition, Futura 75 produced the highest content of *γ*-tocopherol in the middle of maturation, while Carmagnola produced it at the beginning.

Massuela *et al*. [36] reported the effect of harvest time and pruning technique on the total CBD content of a chemotype III medicinal cannabis genotype under indoor cultivation. Over time, the total CBD content did not change significantly, with the maximum total CBD yield found at 9 weeks of flowering. In another report, the total CBD concentration increased in five genotypes of hemp until it reached a peak at flowering, which was 6 to 7 weeks after post-anthesis. Two genotypes showed a concentration plateau until 10 weeks post anthesis, whereas the concentration decreased after the peak plant growth in three genotypes [37]. Burgel *et al.* investigated the impact of growth stage and biomass fractions (inflorescence, upper and lower leaves) for six hemp monoecious genotypes (Fedora 17, Ferimon, Felina 32, Futura 75, Santhica 27, and USO31) and one dioecious (Finola) on cannabinoid content and yield at four stages, consisting of vegetative, bud, full-flowering, and seed maturity [9]. The results of this study demonstrated that cannabinoid production is highly dependent on the genotype and the developmental stage of the plant. Burgel *et al.* [9] did not investigate stem residues from Finola, which is an important by-product of seed production that has received little attention in the literature. In addition, few studies reported the compositional changes in a variety of natural products from the threshing residues at different stages of hemp maturation.

Hemp harvest times can have a significant impact on the chemical composition. Therefore, this work investigates the compositional changes in lipids (waxes), essential oils, and cannabinoids, at different stages of hemp maturation for the inflorescences (tops), leaves, and, importantly, stems of the Finola genotype. This variety was selected as it is bred for high seed oil production, thus resulting in a variety of threshing residues that warrant valorization. The lipophilic molecules found in the different threshing residues were characterized, and the compositional changes in essential oils, cannabinoids, and waxes were investigated to determine the optimum harvesting stage for the different classes of compounds.

## 2. Material and methods

### 2.1. Plant material

**Figure 1.**
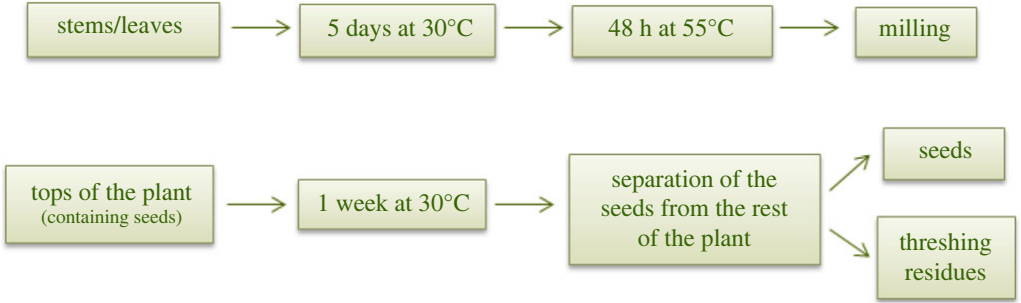
Drying and sample preparation for the stems, leaves, and tops.

A total of 160 hemp seeds (Finola variety) were sowed and placed in a growing chamber. Two weeks after seeding, the plants were re-potted. Some plants germinated later than others and were therefore re-potted after a further 4 days. Once the male and female plants were distinguishable, the former were removed and discarded while the latter were kept. A total of 90 female plants were available for the study. The plants were harvested at different stages as follows (18 female plants were collected for each harvest): (i) harvest 1 (vegetative stage, four weeks after seeding); (ii) harvest 2 (bud stage, eight weeks after seeding); (iii) harvest 3 (full-flowering stage, nine weeks after seeding); and (iv) harvest 4 (seed maturity stage, 11 weeks after seeding). During harvesting, the plants were separated into the leaves, stems, and tops. Two different procedures for drying and sample preparation were carried out, as shown in figure 1. Separation of seeds from the tops was carried out using Seed Processing Holland apparatus.

### 2.2. Soxhlet extraction

Approximately 10 g of milled biomass (hemp leaves, stems, or tops) was placed in a Soxhlet thimble, which was inserted into the Soxhlet apparatus. This was fitted with a 250 ml round-bottom flask containing heptane (200 ml) or acetone (200 ml). A Radleys Discovery Technologies 2006T thermocouple was used to monitor the temperature during the extraction. The solution was refluxed for 4 h. When the extraction was finished, the solvent was removed *in vacuo*. The samples were further dried at room temperature for 24 h before weighing to ensure the removal of traces of residual solvent. The crude wax product was weighed and the percentage yield calculated (w/w of biomass). For each biomass (stems, leaves, or tops), three extractions were carried out and an average percentage yield calculated.

### 2.3. Derivatization prior to high temperature gas chromatography (HTGC) analysis

The silylated derivatization of samples was carried out in the same manner as previously reported [6]. Briefly, 25 mg of crude lipid extract was silylated by addition of 200 µl *N*,*O*-bis-(trimethylsilyl)-trifluoro-acetamide (BSTFA) and 100 µl toluene. The solution was heated for 45 min at 75°C. BSTFA is the best option for analysing a wide range of polar organic compounds because it has a high conversion efficiency, does not require purification, and the derivatives can be injected directly into the GC system. However, it also has some disadvantages, such as the volatility, flammability, and moisture sensitivity of the silylation reagent, in addition to it being an irritant to eyes, skin, and the respiratory system [38].

### 2.4. High temperature gas chromatography (HTGC) method for analysis of waxes

HTGC analysis was performed on an Agilent Technologies 6890N Network GC System. A ZB-5HT capillary column (30 m × 250 µm × 0.25 µm nominal) was fitted at constant pressure of 154 kPa. The carrier gas used was helium. The injector temperature and the flame ionization detector temperature were maintained at 300°C. The samples were injected by automated injection (1 µl injection volume) with a split ratio of 5 : 1. An initial oven temperature of 60°C was maintained for 1 min. The temperature was increased at a ramp rate of 8°C min^−1^ until 360°C and held at this temperature for 30 min.

The samples were quantified by using internal standard calibration and response factor (R*_f_*), with tetradecane as the internal standard. The mass ratios of the samples were plotted against the area ratios in six-point linear calibration curves using external standards for the quantification of hydrophobic molecules (fatty acids, alkanes, alcohols, aldehydes, sterols, triterpenes, and wax esters), where the area ratios were plotted against the mass ratios of the six samples. The following equation was used to compute the *R*_f_ values for each external standard:

$$\frac{{\mathrm{Mass}}_{\mathrm{Product}}}{{\mathrm{Mass}}_{\mathrm{Standard}}}=R_{f}\;\times \;\frac{{\mathrm{Area}}_{\mathrm{Product}}}{{\mathrm{Area}}_{\mathrm{Standard}}}.$$

Silylated calibration curves and *R*_f_ were also generated for the silylated compound groups (fatty acid, alcohols, sterols, and cannabinoids). The signal-to-noise method was used to determine the limit of detection (LOD) and quantification (LOQ). The peak-to-peak noise (N) around the standard analyte retention time was measured, and then the quantity of the standard analyte that would yield a signal (S) equal to a certain value of S/N was estimated. S/N = 3 is commonly used to calculate the LOD, while S/N = 10 is used to calculate the LOQ [39].

### 2.5. High temperature gas chromatography mass spectrometry (HTGC-MS) procedure for analysis of wax

HTGC-MS was performed on a Perkin Elmer Clarus 500 GC coupled with a Clarus 500 quadrupole mass spectrometer. This was fitted with a DB5HT capillary column (30 m × 250 µm × 0.25 µm nominal) at constant pressure of 154 kPa. The carrier gas used was helium. The temperature of the injector was 300°C and the flow rate was set to 1.2 ml min^−1^. The initial oven temperature was maintained at 60°C for 1 min. The temperature was then ramped at a rate of 8°C min^−1^ until 360°C and held for 10 min. The Clarus 500 quadrupole mass spectra were operated in the electron ionization mode (EI) at 70 eV, with a source temperature of 300°C, and the quadrupole set at the scan range of 30–1200 amu per second.

Another method was developed for the analysis of wax esters. The temperature of the injector was 380°C and the flow rate was set to 1.2 ml min^−1^. The initial oven temperature was maintained at 100°C for 1 min. The temperature was then ramped at a rate of 10°C min^−1^ until 380°C and held for 20 min. The Clarus 500 quadrupole mass spectra were operated in the electron ionization mode (EI) at 70 eV, with a source temperature of 300°C, and the quadrupole set at the scan range of 30–1200 amu per second. The data was processed with PerkinElmer enhanced TurboMass (v. 5.4.2) chemical software and compounds were identified by analysing the mass fragmentation patterns, comparison of mass fragmentation patterns with spectra contained in the NIST library (v. 2.2), and direct comparison with standard compounds.

## 3. Results and discussion

### 3.1. Yield obtained for the tops (threshing residues), stems, and leaves

**Figure 2.**
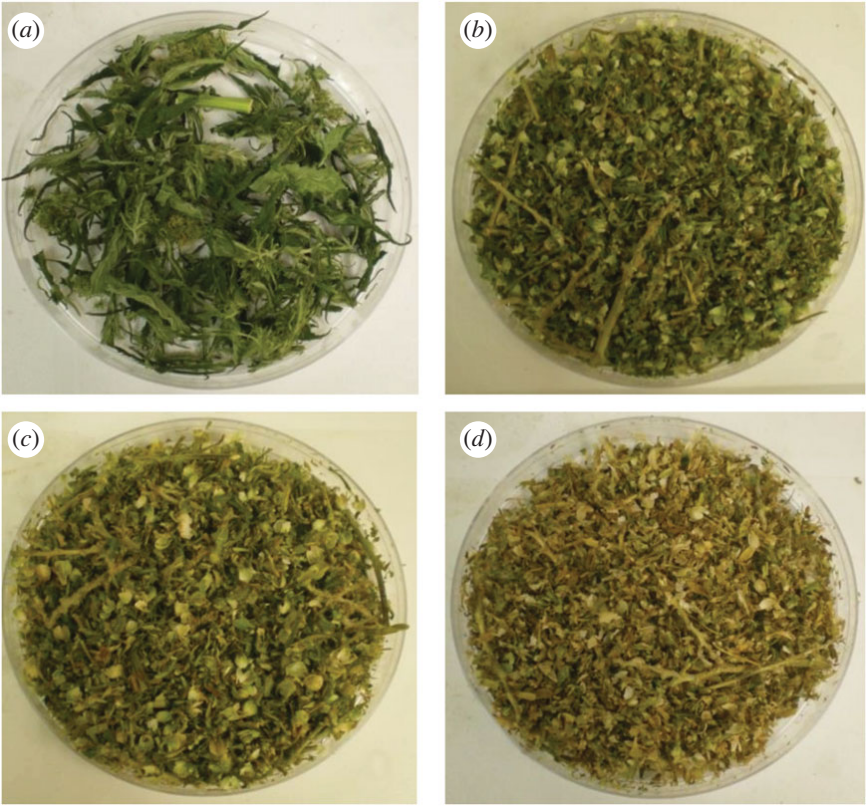
Threshing residues (tops) obtained after four harvests.

The first harvest was carried out at the vegetative stage, which corresponds to the time of harvest for the hemp fibre varieties. At this stage, low amounts of tops were observed (figure 2) when compared with the other harvests. Table 1 summarizes the amounts of threshing residues for each harvest; these increased from the first harvest to the fourth harvest due to growth of the plant. The tops collected from the second, third, and fourth harvesting stages had a characteristic odour indicating the presence of essential oils. Between the third and fourth harvesting stages, the chlorophyll began to degrade leading to the change in colour observed in the threshing residues from the fourth harvest.

**Table 1.** Amounts of threshing residues for each harvest.

harvest	1 (vegetative stage)	2 (bud stage)	3 (flowering stage)	4 (seed maturation)
amount of threshing residue (g)	2.8	30.4	32.5	38.8

There was an increase in the volume of leaves cultivated with each harvesting stage (from 2.32% yield in the first harvest to 3.91% yield in the fourth harvest). However, they became drier and brown in colour after the second harvest due to the degradation of chlorophyll. The stems became longer, drier, and stronger with time (due to the formation of lignin in the stems).

The threshing residues from the tops contained greater amounts of essential oils, while the stems and leaves had a higher wax content. Therefore, for this reason, Soxhlet extractions were carried out using acetone for the threshing residues and heptane for the stems and leaves. Table 2 summarizes the average yields obtained for the various parts of the plants at different harvesting stages. The increase in the average dry weight of inflorescences was similar to previously reported [36]. A similar trend was observed for the threshing residues (tops), with an increase in crude yield with time.

**Table 2.** Yields of extractives for the different parts of the plants at different stages of growth (Soxhlet extraction with heptane for the extraction of leaves and stems, and acetone for the tops of the plant).

harvest	stems (%)	leaves (%)	tops (%)
1 (vegetative stage)	1.52	2.32	3.22
2 (bud stage)	0.99	3.84	6.33
3 (flowering stage)	0.73	3.82	6.40
4 (seed maturation stage)	0.67	3.91	6.47

### 3.2. Compositional changes in stems, leaves, and tops with time

**Figure 3.**
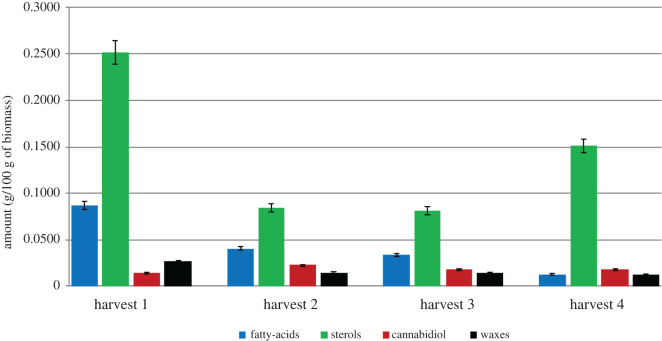
Compositional change (g/100 g of biomass) of major classes of compounds found in the stems with time.

**Figure 4.**
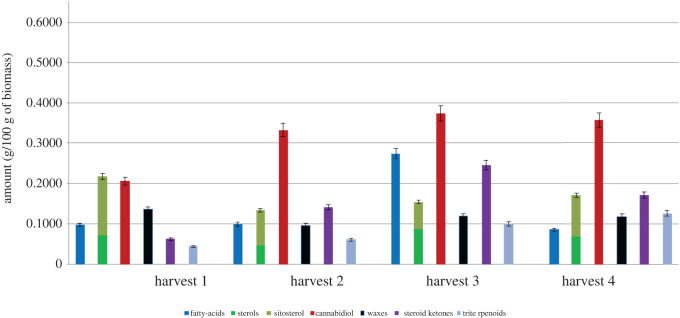
Compositional change (g/100 g of biomass) of major classes of compounds found in the leaves with time.

**Figure 5.**
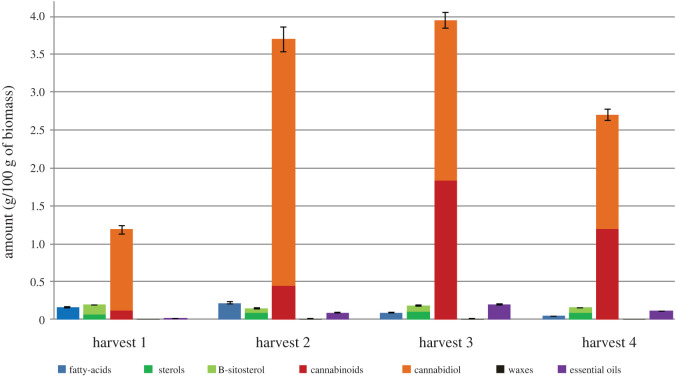
Compositional change (g/100 g of biomass) of major classes of compounds found in the tops with time.

To investigate the compositional changes observed at the different harvesting stages, the major compounds were identified by GC-MS and the use of standards. However, quantification of the compositional changes at the different harvesting stages was conducted using GC-FID, as presented in tables 3–5. Figures 3–5 summarize the major compounds identified in the stems, leaves, and tops after each harvesting cycle. The LOD and LOQ found for the analyte standards ranged from 10.3 to 155.9 µg g^−1^ of biomass and from 34.3 to 519.7 µg g^−1^ of biomass, respectively (electronic supplementary material, table S2). The precision of the analytical method was assessed in relation to the levels of repeatability by estimating the relative standard deviation (RSD) for each compound analysed from successive measurements. The RSD values ranged from 0.2 to 2.0% and did not exceed the acceptance limit of 15% established by the US FDA (electronic supplementary material, table S2) [40].

**Table 3.** Quantification of the compounds in the Finola stems**.**

	harvest 1		harvest 2		harvest 3		harvest 4	
	mass in 100 g of wax (g)	mass in 100 g of biomass (g)	mass in 100 g of wax (g)	mass in 100 g of biomass (g)	mass in 100 g of wax (g)	mass in 100 g of biomass (g)	mass in 100 g of wax (g)	mass in 100 g of biomass (g)
palmitic acid	4.1013	0.0623	2.7638	0.0274	3.2769	0.0239	1.1924	0.0080
linoleic acid	0.5794	0.0088	0.1552	0.0015	0.1692	0.0012	0.2707	0.0018
linolenic acid/oleic acid	0.8025	0.0122	1.0900	0.0108	1.0365	0.0076	0.2261	0.0015
stearic acid	0.2218	0.0034	0.1319	0.0013	0.1264	0.0009	0.2307	0.0015
heptacosane	0.2644	0.0040	0.1394	0.0014	0.3051	0.0022	0.1999	0.0013
nonacosane	0.4039	0.0061	0.3656	0.0036	0.4768	0.0035	0.5963	0.0040
hentriacontane	0.0840	0.0013	0.1414	0.0014	0.0718	0.0005	0.2647	0.0018
tricosanal	0.2067	0.0031	1.4571	0.0144	0.8454	0.0062	1.0265	0.0069
octacosanal	0.2552	0.0039	0.2659	0.0026	0.2210	0.0016	0.7734	0.0052
campesterol	3.8770	0.0589	1.5620	0.0155	2.2069	0.0161	5.2735	0.0353
stigmasterol	2.2035	0.0335	1.1825	0.0117	1.4010	0.0102	2.7922	0.0187
*β*-sitosterol	10.4886	0.1594	5.7764	0.0572	7.5055	0.0548	14.4753	0.0970
*β*-amyrin	1.4622	0.0222	1.3499	0.0134	1.3703	0.0100	2.1754	0.0146
α-amyrin	0.3810	0.0058	0.4246	0.0042	0.6857	0.0050	1.6516	0.0111
stigmast-4-en-3-one	1.3656	0.0208	1.3589	0.0135	1.5216	0.0111	2.1461	0.0144
stigmasta-3,5-diene	0.5879	0.0089	0.5770	0.0057	0.6011	0.0044	0.2182	0.0015
cannabidiol	0.9165	0.0139	2.3145	0.0229	2.4556	0.0179	2.6858	0.0180
THC	0.4841	0.0074	0.2423	0.0024	0.3309	0.0024	0.3221	0.0022
octacosanol	0.5903	0.0090	0.5224	0.0052	0.7322	0.0053	0.4149	0.0028
wax C40	0.2689	0.0041	0.3234	0.0032	0.3159	0.0023	0.3090	0.0021
wax C42	0.2803	0.0043	0.3040	0.0030	0.2816	0.0021	0.3346	0.0022
wax C44	0.3753	0.0057	0.3449	0.0034	0.3608	0.0026	0.4021	0.0027
wax C46	0.3506	0.0053	0.2427	0.0024	0.3525	0.0026	0.3139	0.0021
wax C48	0.2368	0.0036	0.1673	0.0017	0.3280	0.0024	0.2688	0.0018
wax C50	0.2368	0.0036	0.1177	0.0012	0.2656	0.0019	0.1585	0.0011
total	31.0244	0.4716	23.3209	0.2309	27.2445	0.1989	38.7229	0.2594

**Table 4.** Quantification of the compounds in the Finola leaves.

	harvest 1		harvest 2		harvest 3		harvest 4	
	mass in 100 g of wax (g)	mass in 100 g of biomass (g)	mass in 100 g of wax (g)	mass in 100 g of biomass (g)	mass in 100 g of wax (g)	mass in 100 g of biomass (g)	mass in 100 g of wax (g)	mass in 100 g of biomass (g)
palmitic acid	1.297	0.030	1.275	0.049	2.372	0.091	0.967	0.038
linoleic acid/oleic acid	2.459	0.057	1.010	0.039	3.995	0.153	1.079	0.042
stearic acid	0.439	0.010	0.272	0.010	0.799	0.031	0.126	0.005
heptacosane	0.917	0.021	0.158	0.006	0.116	0.004	0.463	0.018
nonacosane	2.846	0.066	2.041	0.078	1.901	0.073	2.335	0.092
hentriacontane	0.076	0.002	0.471	0.018	0.471	0.018	0.658	0.026
tricosanal	0.723	0.017	0.439	0.017	0.549	0.021	0.760	0.030
campesterol	2.123	0.049	0.632	0.024	2.087	0.080	0.850	0.033
stigmasterol	0.979	0.023	0.566	0.022	0.169	0.006	0.871	0.034
*β*-sitosterol	6.245	0.145	2.285	0.088	1.770	0.068	2.620	0.103
*β*-amyrin	1.341	0.031	0.821	0.032	1.816	0.069	2.155	0.085
α-amyrin	0.551	0.013	0.730	0.028	0.782	0.030	1.061	0.042
stigmast-4-en-3-one	1.846	0.043	1.635	0.063	3.020	0.115	2.254	0.089
stigmastane-3,6-dione	0.813	0.019	2.018	0.078	3.412	0.130	2.111	0.083
cannabidiol	8.837	0.205	8.665	0.333	9.784	0.374	9.089	0.357
THC	1.569	0.036	0.986	0.038	1.629	0.062	0.432	0.017
2-pentadecanone-6,10,14-trimethyl	1.251	0.029	1.327	0.051	0.554	0.021	0.363	0.014
squalene	0.241	0.006	0.420	0.016	0.092	0.004	0.140	0.006
*α*-tocopherol	2.216	0.051	2.067	0.079	2.265	0.087	1.532	0.060
*γ*-tocopherol	0.675	0.016	1.373	0.053	0.326	0.012	0.939	0.037
wax C40	0.952	0.022	0.462	0.018	0.517	0.020	0.421	0.017
wax C41	0.072	0.002	0.032	0.001	0.041	0.002	0.038	0.001
wax C42	1.446	0.034	0.590	0.023	0.688	0.026	0.684	0.027
wax C43	0.113	0.003	0.063	0.002	0.079	0.003	0.065	0.003
wax C44	1.479	0.034	0.559	0.021	0.687	0.026	0.691	0.027
wax C45	0.098	0.002	0.050	0.002	0.096	0.004	0.066	0.003
wax C46	0.825	0.019	0.352	0.013	0.465	0.018	0.491	0.019
wax C47	0.153	0.004	0.053	0.002	0.081	0.003	0.057	0.002
wax C48	0.396	0.009	0.212	0.008	0.265	0.010	0.281	0.011
wax C49	0.098	0.002	0.030	0.001	0.051	0.002	0.049	0.002
wax C50	0.189	0.004	0.120	0.005	0.158	0.006	0.167	0.007
total	43 267	1004	31 712	1218	41 039	1568	33 816	1329

**Table 5.** Quantification of the compounds in the tops of Finola (g/100 g of threshing residues).

	harvest 1	harvest 2	harvest 3	harvest 4
palmitic acid	0.0137	0.0243	0.0139	0.0052
linoleic acid/oleic acid	0.1198	0.1237	0.0656	0.0322
stearic acid	0.0295	0.0701	0.0106	0.0100
tetracosane	0.0144	0.0088	0.0089	0.0060
octacosane	0.0103	0.0060	0.0063	0.0048
nonacosane	0.0378	0.0863	0.0841	0.0583
triacontane	0.0045	0.0074	0.0074	0.0048
hentriacontane	0.0059	0.0107	0.0125	0.0082
*β*-tocopherol	0.0072	0.0175	0.0196	0.0123
*α*-tocopherol	0.0142	0.0222	0.0293	0.0147
campesterol	0.0206	0.0156	0.0180	0.0126
stigmasterol	0.0177	0.0143	0.0124	0.0102
*β*-sitosterol	0.1235	0.0474	0.0779	0.0605
*β*-amyrin	0.0206	0.0465	0.0576	0.0527
α-amyrin	0.0119	0.0206	0.0237	0.0192
12-olean-3-yl-acetate	0.0071	0.0394	0.0474	0.0352
9,19-cyclolanost-24-em-3-ol-acetate	0.0216	0.0213	0.0249	0.0104
tetrahydrocannabivarin	0.0065	0.0108	0.0443	0.0106
cannabigerol	0.0784	0.2737	0.6106	0.0812
cannabidiol	1.0649	3.2515	2.1161	1.4997
THC	0.0106	0.0157	0.9873	0.9826
cannabinol	0.0259	0.1441	0.1892	0.1228
hexacosanol	0.0249	0.0174	0.0171	0.0100
octacosanol	0.0057	0.0169	0.0180	0.0148
wax C40	0.0073	0.0100	0.0088	0.0038
wax C42	0.0011	0.0033	0.0052	0.0027
wax C44	0.0000	0.0021	0.0029	0.0016
total	17 058	43 276	45 201	30 869

#### 3.2.1. Stems

The major compounds identified in the stems were sterols (mainly *β*-sitosterol, campesterol, and stigmasterol), saturated and unsaturated fatty-acids (palmitic acid, linoleic acid, *α*-linolenic acid, oleic acid, and stearic acid), CBD, and wax esters, as shown in figure 3. Small amounts of tetrahydrocannabinol (THC) (less than 0.2%) were also detected. The profiles of lipid in both stems and leaves were comparable to hemp dust wastes produced during the production of fibres [6].

The first harvest contained the largest quantities of total compounds (4716 µg g^−1^ of biomass; table 3), which correlates with the crude yield data previously shown. Figure 3 shows the variation in the quantities of the major families of compounds with time. There is a significant difference in the wax content between the stems and leaves at different stages of growth (harvesting stages). With regard to the stems, the amount of wax decreases over time from the first harvest (266 µg g^−1^ of biomass) to the fourth harvest (120 µg g^−1^ of biomass) (table 3). By contrast, the amounts of waxes found in the leaves increases with time. Images of the extracts from the leaves obtained from the different harvesting stages may be viewed in the supplementary information (electronic supplementary material, figure S2). A similar trend was observed for the threshing residues (tops), with an increase in crude yield with time.

For all harvesting periods, the dominant group of compounds found in the stems were the phytosterols. By far, the largest amount of phytosterols were observed in the stems obtained from the first harvest, with *β*-sitosterol found especially in high quantities (1594 µg g^−1^ of biomass; table 3). This result was consistent with previous studies of Futura 75 and Carmagnola hemp varieties that indicated the most phytosterol was *β*-sitosterol [35]. A decrease in phytosterol content was observed between the first harvest and the third harvest (flowering); this pattern was also observed in Carmagnola [35].

According to a recent study on secondary metabolites in cannabis, stem bark showed a high sterol content, with *β*-sitosterol as the most abundant while cannabinoid was found in small amounts [41]. The benefits of phytosterols have been well-established in the literature and these compounds are found in numerous food and cosmetic products. Sterols have been proven to lower cholesterol levels in the blood and studies have shown that they are also effective anti-cancer compounds. With respect to the saturated and unsaturated fatty acids and wax esters, it can be noted that the amounts decrease with an extended time of harvest. The amounts of cannabinoids in the stems were found to be very low for all harvesting periods. Considering the cannabinoid content from the stems, it was lower compared with the tops and the growth stage of the plant had only a minor influence on the cannabinoid content (around 202–253 µg g^−1^ of biomass for different harvest stages), with a slight increase at the second harvest stage (bud stage, 253 µg g^−1^ of biomass). This observation is consistent with the reports from Mastellone *et al*. that indicated the cannabinoid content from the stem of *Cannabis sativa* L. was independent of the growth stage of harvesting and the content showed a slight increase at the stage closer to flowering [11]. Free fatty acid (FFA) composition in the hemp was consistent with previous studies [42], which highlighted that palmitic acid was the most abundant FFA, followed by oleic acid and stearic acid. In addition, the compositional changes of FFA during the growth stage are consistent with previously reported studies [43], which indicated a decrease in FFA content with increasing stage of growth.

#### 3.2.2. Leaves

Significantly, larger quantities of lipophilic molecules were found in the leaves compared with the stems for all harvesting periods. This was expected as the leaves have a higher surface area compared with the stems and are therefore more prone to loss of water via transpiration, and therefore require larger quantities of wax on their surface. Figure 4 shows that the greatest abundance of total compounds was found in the leaves that were collected at the third harvest (15 680 µg g^−1^ of biomass; table 4). A wider family of compounds was found in the leaves compared with the stems, with the presence of steroid ketones and triterpenoids in addition to the groups of compounds found within the stem. Considerably high quantities of cannabinoids were identified in the leaves of Finola, with CBD being the major cannabinoid detected (THC was detected, albeit in small amounts of less than 0.2%). The amount of CBD increases with time. The largest quantities of CBD were found in the leaves obtained from the third harvest (3740 µg g^−1^ of biomass; table 4), albeit similar quantities were also found in the second and fourth harvests (3330 and 3570 µg g^−1^ of biomass, respectively; table 4). Similar results for the CBD content from leaves of the Finola variety were demonstrated by Burgel *et al.* [9]. They reported the highest CBD content was recorded in leaves of the Finola genotype at the full-flowering stage and the content decreased at the seed maturity stage. However, the CBD content was not determined at either the vegetative or bud stages of growth. The current work was able to detect CBD in the leaves even at early stages of plant growth: 2050 µg g^−1^ of biomass at the vegetative stage (harvest 1) and 3330 µg g^−1^ of biomass at the bud stage (harvest 2), as shown in table 4. Aizpurua-Olaizola *et al*. [44] also reported similar results for the CBD content in hemp leaves and they indicated that it was low in the vegetative stage, followed by an increase in the flowering stage due to the decarboxylation of the cannabidiolic acid (CBDA). Moreover, the development of THC content in the hemp leaves from this work during plant growth was also similar to the reports from the same group [44], as is the case of the development of CBD content during hemp growth.

Interestingly, the quantities of phytosterols in the leaves followed the same pattern as found in the stem, with the largest amount of phytosterol found in the leaves obtained from the first harvest (2170 µg g^−1^ of biomass; table 4). Significant quantities of steroid ketones were found in the leaves, which increased over time until a maximum was reached at the third harvest (2450 µg g^−1^ of biomass; table 4)—this is interesting as it is the reverse to what was seen with the sterol compounds. This suggests that with time, the sterols are converted to steroid ketones. Two triterpenoids were found in the extracts, *α*- and *β*-amyrin, which increased in concentration until the final harvest (1270 µg g^−1^ of biomass; table 4). These compounds have pharmacological activities in several systems such as the central and peripheral nervous systems, gastrointestinal tract, and immunological system, with several effects such as anti-inflammatory, antidepressive, anticonvultant, hepato-protective, gastroprotective, anticholytic, antipancreatitic, antihyperglycermic, and hypolipidemic effects [45]. As expected, the leaves contained the largest quantities of wax esters (compared with the stem and the tops), with similar quantities found in the leaves from each harvest. Wax esters are desirable due to their high molecular weight, which enables them to be used in various applications, including coatings, polishes, cosmetics, plasticizers, and lubricants. Saturated and unsaturated fatty acids were present in similar amounts to the fatty acids found in the stem; however, their amounts increased over time and reached the maximum at the third harvest, which was in stark contrast to their changes in the stem. This is consistent with a previous study that demonstrated the ratio of polyunsaturated to saturated fatty acid of Futura 75 and Carmagnola increased during ripening of the plants [35]. In addition, Futura 75 produced the highest content of *γ*-tocopherol during the middle of maturation, which is consistent with the results observed in this current study [35].

#### 3.2.3. Tops

Figure 5 summarizes the different groups of compounds identified in the tops. Like the leaves, the greatest abundance of total compounds was found in the tops obtained from the third harvest (47 231 µg g^−1^ of biomass; table 5 and figure 5). The extracts are dominated by the cannabinoids, which form approximately 85% of the total composition of the extracts obtained from the second, third, and fourth harvests. The largest quantities of total cannabinoids were found in the extracts obtained from the tops of the third harvest (39 475 µg g^−1^ of biomass; table 5 and figure 5) and the amounts of cannabinoids decreased at the final harvest stage. This trend contradicted the findings of Burgel *et al.* who identified no significant differences in the CBD content between the full-flowering and seed maturity stages [9]. However, it was comparable to results found by Mussuela *et al*. and Aizpurua-Olaizola *et al*. for different genotypes of hemp [36,44]. As expected, a wider variety of cannabinoids were detected in the tops, which is comparable with previous experiments [41]. The major cannabinoid present is CBD, which is found in the largest quantities in the tops collected at the second harvest (32 515 µg g^−1^ of biomass; table 5). However, previous research reported the highest CBD content from inflorescence of the Finola variety at the seed maturity stage [9]. However, the Ferimon variety had the same trend reported for CBD, which increased from the first harvest stage (vegetative growth) to reach the maximum at the second harvest stage (bud) and decreased over the third and fourth harvest stages (flowering and seed maturity, respectively) [9]. Similarly, CBD content increased with plant age, reaching the maximum level at the budding stage [46]. Other cannabinoids found were THC, cannabigerol (CBG), tetrahydrocannabivarin (THCV), and cannabinol (CBN). There was a significant increase (63%) in the level of THC content when moving from the second harvest (157 µg g^−1^ of biomass) to the third harvest (9873 µg g^−1^ of biomass), as shown in table 5. This is quite significant in terms of harvesting; since THC is a banned substance in consumer products, it would be preferable to harvest the tops after the second harvest (higher CBD content), albeit there were higher concentrations of cannabinoids after the third harvest at week 9. Besides, in hot and dry conditions, the late stage of growth may sometimes result in THC levels over 0.2%; as such, harvesting at the earlier stage of growth is preferable [47]. This would give a higher CBD:THC ratio, which would make purification of CBD via short-path distillation and crystallization significantly easier. CBG, the first cannabinoid synthesized, was found in the tops (inflorescences), which is in contrast to studies of Burgel *et al*. [9] where no CBG content was observed in the Finola genotype and it was only found in the Santica75 genotype. In addition, Glivar *et al.* [48] also did not detect CBG in the Finola variety. However, the development of CBG content in hemp inflorescences of the Finola genotype grown in this study demonstrated the same trend as previously reported studies, i.e. the CBG content increased to reach a maximum at the third harvest stage followed by decreasing at the late stage due to decarboxylation of cannabigerolic acid during the growth of the plant [44]. Importantly, CBG has the potential to be used to treat various diseases such as glaucoma, inflammatory bowel disease, and prostate cancer [44]. Madaka *et al.* [49] reported the purification of this class of compounds from *Cannabis sativa* L. The use of supercritical fluid extraction (SFE) and flash chromatography in the RevelerisPREP purification system led to the isolation and purification of three cannabinoids including CBD, CBN, and THC with a high purity. Recently, Liu *et al*. [50] reported an extensive review of various advanced technologies for extracting, separating, purifying, and identifying the bioactive compounds in *Cannabis sativa*. The separation and purification of phytochemicals from this plant have been reported using solid-phase extraction (SPE), centrifugal partition chromatography (CPC), preparative high-performance liquid chromatography (prep-HPLC), and hydrophilic interaction liquid chromatography (HILIC). Such processes may find industrial relevance in the future for the isolation of these important biologically active compounds.

Once again, it is interesting to note that *β*-sitosterol is found in the largest quantities in the tops collected from the first harvest (1235 µg g^−1^ of biomass; table 5), which correlates with what was observed in the stems and the leaves. The first harvest also gave rise to the largest amounts of total sterols (1618 µg g^−1^ of biomass; table 5). This indicates that the phytosterols are present in the largest quantities during the initial development of the plant.

Besides waxes, several essential oils were present in the extracts from the tops. GC-FID and GC-MS data showed that the major essential oil compounds found in the extracts from the tops were *α*-pinene, *β*-pinene, myrcene, *d*-limonene, *β*-ocimene, terpinolene, *β*-caryophyllene, and *α*-caryophyllene, while minimal quantities of *α*-bergamotene, *β*-selinene, and caryophyllene oxide were also detected (electronic supplementary material, figure S7). The essential oil composition followed the same trend as the cannabinoid composition, i.e. there was an increase in the essential oil composition with time up until the third harvest (2030 µg g^−1^ of biomass), following which a decrease was observed in the fourth harvest. The observed results are consistent with previously reported studies of the essential oil content from inflorescences of the Fedora 17 variety, in which the later harvest shows lower yields of extraction compared with the earlier harvests. As such, the harvest time plays a significant role on the essential oil content and therefore the hemp tops should be harvested before their full maturity [33]. However, this contrasts with the reports of Abdollahi *et al*. that demonstrated essential oils were unaffected by the growth stage and the differences observed were likely to relate to the genotype of hemp [34]. The essential oil of hemp is considered as a product with high added value, which can be used in cosmetics for soap, shampoo, perfume, and cream productions, as well as in food as additives in bakery and catering, or as a flavouring for alcoholic and non-alcoholic beverages. Essential oils are also used in topical treatments of wounds and skin infections. Furthermore, essential oils have also been used in crop protection as an alternative to petrochemical-derived products [51]. Significant decreases in the amounts of terpenoids are observed from the top to bottom of the inflorescences from *Cannabis sativa*; similar observations have also been made for cannabinoids. Moreover, it has been proposed that even trace amounts of terpenes can have a significant impact on cannabinoid activity [52].

The results obtained in this study are promising as they highlight a potential feedstock of lipophilic molecules. These molecules can find use in a host of applications including nutraceuticals, pharmaceuticals, and food and beverage products, as well as detergents, lubricants etc. Significantly large quantities of cannabinoids are found in the tops and the leaves. Cannabinoids, especially CBD, are highly sought after and significant effort is currently underway to obtain them. CBD has a host of therapeutic and pharmacological properties and is used in the treatment of numerous central nervous and peripheral disorders. Clinical-level studies have been conducted whereby its therapeutic efficacy has been demonstrated. The first phytocannabinoid drug, Savitex, was approved in 2011 in the UK for treating multiple sclerosis spasms. Furthermore, a recent study has shown the promise of using CBD in the treatment of viral hepatitis C, which currently has no vaccine. Therefore, this study is important as it not only highlights which threshing residues contain CBD but also highlights the harvesting period to obtain maximum quantities of valuable molecules. Furthermore, it indicates the optimum harvesting period in terms of the purity of CBD, as significant quantities of other undesired cannabinoids (e.g. THC) significantly increase over time but are present in only small quantities at the early stages of plant growth.

## 4. Conclusion

In this work, the compositional changes in several major classes of compounds (fatty acids, alkanes, sterols, wax esters, and cannabinoids) as a function of the plant's growth have been investigated. Such data is vital for maximizing the value of natural products such as the cannabinoids from waste threshing residues. The highest crude yield of extracts was found to be at the first harvesting stage (four weeks after seeding) for the stems (1.52%), while for the leaves and tops, the fourth harvesting stage (11 weeks after seeding) gave the highest crude yields (3.91% and 6.47%, respectively). The greatest number of cannabinoids was found in the extracts from the tops (constituting over 85% of the total extracts), while the leaves had the largest quantities of wax esters and steroid compounds. The distillate of CBD is valued at $3000 kg CBD^−1^ and the isolate is valued at $1000 kg CBD^−1^. In the tops, there was an increase in the essential oil and cannabinoid content with time, up until the third harvest. This was followed by a decrease in their quantities at the final harvest. Although the highest quantity of CBD was found in the third week of harvest, the best CBD:THC ratio was seen after the second harvest, with quantities of THC being significantly lower than at the third harvest—this would make isolation and purification of CBD much easier. Interestingly, for the stem, leaf, and top extracts, the largest quantities of *β*-sitosterol were found in the stems, leaves, and tops obtained from the first harvest stage. The high market price for cannabinoids such as CBD may result in a shift in hemp production from seed or fibres to this valuable natural product with potential to be used in the development of natural consumer products. This study highlights the importance of discerning the variation in the composition of compounds such as CBD with time within the plant to ascertain an adequate harvesting period to maximize these industrial products.

## Data Availability

The datasets supporting this article have been uploaded as part of the electronic supplementary material [53].

## References

[1] Nissen L, Zatta A, Stefanini I, Grandi S, Sgorbati B, Biavati B, Monti A. 2010 Characterization and antimicrobial activity of essential oils of industrial hemp varieties (Cannabis sativa L. Fitoterapia **81**, 413-419. (10.1016/j.fitote.2009.11.010) 19969046

[2] Kreuger E, Prade T, Escobar F, Svensson S-E, Englund J-E, Bjornsson L. 2011 Anaerobic digestion of industrial hemp–Effect of harvest time on methane energy yield per hectare. Biomass Bioenerg. **35**, 893-900. (10.1016/j.biombioe.2010.11.005)

[3] Rehman MSU, Rashid N, Saif A, Mahmood T, Han J-I. 2013 Potential of bioenergy production from industrial hemp (Cannabis sativa): Pakistan perspective. Renew. Sust. Energ. Rev. **18**, 154-164. (10.1016/j.rser.2012.10.019)

[4] Da Porto C, Decorti D, Tubaro F. 2012 Fatty acid composition and oxidation stability of hemp (*Cannabis sativa* L.) seed oil extracted by supercritical carbon dioxide. Ind. Crops Prod. **36**, 401-404. (10.1016/j.indcrop.2011.09.015)

[5] Teh S-S, Birch J. 2013 Physicochemical and quality characteristics of cold-pressed hemp, flax and canola seed oils. J. Food Compost. Anal. **30**, 26-31. (10.1016/j.jfca.2013.01.004)

[6] Attard TM, Bainier C, Reinaud M, Lanot A, McQueen-Mason SJ, Hunt AJ. 2018 Utilisation of supercritical fluids for the effective extraction of waxes and Cannabidiol (CBD) from hemp wastes. Ind. Crops Prod. **112**, 38-46. (10.1016/j.indcrop.2017.10.045)

[7] Callaway JC. 2004 Hemp seed production in Finland. J Ind. Hemp **9**, 97-103. (10.1300/J237v09n01_11)

[8] Cherney JH, Small E. 2016 Industrial femp in North America: Production, politics and potential. Agronomy **6**, 58. (10.3390/agronomy6040058)

[9] Burgel L, Hartung J, Pflugfelder A, Graeff-Hönninger S. 2020 Impact of growth stage and biomass fractions on cannabinoid content and yield of different hemp (*Cannabis sativa* L.) genotypes. Agronomy **10**, 372. (10.3390/agronomy10030372)

[10] Pavlovic R, Panseri S, Giupponi L, Leoni V, Citti C, Cattaneo C, Cavaletto M, Giorgi A. 2019 Phytochemical and Ecological Analysis of Two Varieties of Hemp (*Cannabis sativa* L.) Grown in a Mountain Environment of Italian Alps. Front. Plant Sci. **10**, 1265. (10.3389/fpls.2019.01265) 31708938 PMC6822994

[11] Mastellone G, Marengo A, Sgorbini B, Scaglia F, Capetti F, Gai F, Peiretti PG, Rubiolo P, Cagliero C. 2022 Characterization and Biological Activity of Fiber-Type *Cannabis sativa* L. Aerial Parts at Different Growth Stages. Plants **11**, 419. (10.3390/plants11030419) 35161400 PMC8838183

[12] Behr A, Johnen L. 2009 Myrcene as a natural base chemical in sustainable chemistry: A critical review. ChemSusChem **2**, 1072-1095. (10.1002/cssc.200900186) 20013989

[13] Bradford PG, Awad AB. 2007 Phytosterols as anticancer compounds. Mol. Nutr. Food Res. **51**, 161-170. (10.1002/mnfr.200600164) 17266177

[14] Gill I, Valivety R. 1997 Polyunsaturated fatty acids, part 1: Occurrence, biological activities and applications. Trends Biotechnol. **15**, 401-409. (10.1016/S0167-7799(97)01076-7) 9351284

[15] Gunawan ER, Basri M, Rahman M, Salleh AB, Rahman R. 2005 Study on response surface methodology (RSM) of lipase-catalyzed synthesis of palm-based wax ester. Enzyme Microb. Technol. **37**, 739-744. (10.1016/j.enzmictec.2005.04.010) PMC2211475 17760990

[16] Hill K. 2000 Fats and oils as oleochemical raw materials. Pure Appl. Chem. **72**, 1255-1264. (10.1351/pac200072071255)

[17] Marinangeli CPF, Jones PJH, Kassis AN, Eskin MNA. 2010 Policosanols as nutraceuticals: Fact or fiction. Crit. Rev. Food Sci. Nutr. **50**, 259-267. (10.1080/10408391003626249) 20301014

[18] Schiestl FP, Ayasse M, Paulus HF, Löfstedt C, Hansson BS, Ibarra F, Francke W. 1999 Orchid pollination by sexual swindle. Nature **399**, 421. (10.1038/20829) 10947239

[19] Sjöström E. 1991 Carbohydrate degradation products from alkaline treatment of biomass. Biomass Bioenerg. **1**, 61-64. (10.1016/0961-9534(91)90053-F)

[20] Hemphill JK, Turner JC, Mahlberg PG. 1980 Cannabinoid content of individual plant organs from different geographical strains of *Cannabis sativa* L. J. Nat. Prod. **43**, 112-122. (10.1021/np50007a009)

[21] Bertoli A, Tozzi S, Pistelli L, Angelini LG. 2010 Fibre hemp inflorescences: From crop-residues to essential oil production. Ind. Crops Prod. **32**, 329-337. (10.1016/j.indcrop.2010.05.012)

[22] Mediavilla V. 1998 The production of essential hemp oil in Switzerland. Technol. Ecol. Symp. **24**, 117-118.

[23] Herzi N, Bouajila J, Camy S, Romdhane M, Condoret J-S. 2013 Comparison of different methods for extraction from Tetraclinis articulata: Yield, chemical composition and antioxidant activity. Food Chem. **141**, 3537-3545. (10.1016/j.foodchem.2013.06.065) 23993518

[24] Ozel MZ, Kaymaz H. 2004 Superheated water extraction, steam distillation and Soxhlet extraction of essential oils of Origanum onites. Anal. Bioanal. Chem. **379**, 1127-1133. (10.1007/s00216-004-2671-5) 15179539

[25] Rodriguez-Solana R, Salgado JM, Dominguez JM, Cortés-Dieguez S. 2014 Characterization of fennel extracts and quantification of estragole: Optimization and comparison of accelerated solvent extraction and Soxhlet techniques. Ind. Crops Prod. **52**, 528-536. (10.1016/j.indcrop.2013.11.028)

[26] Saoud AA, Yunus RM, Aziz RA, Rahmat AR. 2005 Study of eucalyptus essential oil acquired by microwave extraction. Acta Hortic **679**, 173-179. (10.17660/ActaHortic.2005.679.21)

[27] Wu H, Shi J, Xue S, Kakuda Y, Wang D, Jiang Y, Ye X, Li Y, Subramanian J. 2011 Essential oil extracted from peach (Prunus persica) Wkernel and its physicochemical and antioxidant properties. LWT-Food Sci. Technol. **44**, 2032-2039. (10.1016/j.lwt.2011.05.012)

[28] Guan W, Li S, Yan R, Tang S, Quan C. 2007 Comparison of essential oils of clove buds extracted with supercritical carbon dioxide and other three traditional extraction methods. Food Chem. **101**, 1558-1564. (10.1016/j.foodchem.2006.04.009)

[29] Nur Ain AH, Zaibunnisa AH, Halimahton Zahrah MS, Norashikin S. 2013 An experimental design approach for the extraction of lemongrass (Cymbopogon citratus) oleoresin using pressurised liquid extraction (PLE). Int. Food Res. J. **20**, 451-455. (10.1016/j.lwt.2008.03.015)

[30] Wong YC, Ahmad-Mudzaqqir MY, Wan-Nurdiyana WA. 2014 Extraction of essential oil from cinnamon (Cinnamomum zeylanicum). Orient. J. Chem. **30**, 37-47. (10.13005/ojc/300105)

[31] Hanamanthagouda MS, Kakkalameli SB, Naik PM, Nagella P, Seetharamareddy HR, Murthy HN. 2010 Essential oils of Lavandula bipinnata and their antimicrobial activities. Food Chem. **118**, 836-839. (10.1016/j.foodchem.2009.05.032)

[32] Palmieri S, Pellegrini M, Ricci A, Compagnone D, Lo Sterzo C. 2020 Chemical composition and antioxidant activity of thyme, hemp and coriander extracts: a comparison study of maceration, Soxhlet, UAE and RSLDE techniques. Foods **9**, 1221. (10.3390/foods9091221) 32887367 PMC7555591

[33] Ascrizzi R, Ceccarini L, Tavarini S, Flamini G, Angelini LG. 2019 Valorisation of hemp inflorescence after seed harvest: cultivation site and harvest time influence agronomic characteristics and essential oil yield and composition. Ind. Crops Prod. **139**, 111541. (10.1016/j.indcrop.2019.111541)

[34] Abdollahi M, Sefidkon F, Calagari M, Mousavi A, Mahomoodally MF. 2020 Impact of four hemp (*Cannabis sativa* L.) varieties and stage of plant growth on yield and composition of essential oils. Ind. Crops Prod. **155**, 112793. (10.1016/j.indcrop.2020.112793)

[35] Marzocchi S, Caboni MF. 2020 Effect of harvesting time on hemp (*Cannabis sativa* L.) seed oil lipid composition. Ital. J. Food Sci. **32**, 1018-1029. (10.22541/au.158354018.86295548)

[36] Crispim MD, Hartung J, Munz S, Erpenbach F, Graeff-Hönninger S. 2022 Impact of harvest time and pruning technique on total CBD concentration and yield of medicinal cannabis. Plants **11**, 140. (10.3390/plants11010140) 35009146 PMC8747189

[37] Yang R, Berthold EC, McCurdy CR, da Silva Benevenute S, Brym ZT, Freeman JH. 2020 Development of cannabinoids in flowers of industrial hemp (*Cannabis sativa* L.): a pilot study. J. Agric. Food Chem **68**, 6058-6064. (10.1021/acs.jafc.0c01211) 32392412

[38] Pietrogrande MC, Bacco D, Mercuriali M. 2010 GC-MS analysis of low-molecular-weight dicarboxylic acids in atmospheric aerosol: comparison between silylation and esterification derivatization procedures. Anal. Bioanal. Chem. **396**, 877-885. (10.1007/s00216-009-3212-z) 19847406

[39] Sanagi MM, Ling SL, Nasir Z, Hermawan D, Ibrahim WAW, Abu Naim A. 2009 Comparison of signal-to-noise, blank determination, and linear regression methods for the estimation of detection and quantification limits for volatile organic compounds by gas chromatography. J. AOAC Int. **92**, 1833-1838. (10.1093/jaoac/92.6.1833) 20166602

[40] Tiwari G, Tiwari R. 2010 Bioanalytical method validation: an updated review. Pharm. Methods **1**, 25-38. (10.4103/2229-4708.72226) 23781413 PMC3658022

[41] Jin D, Dai K, Xie Z, Chen J. 2020 Secondary metabolites profiled in Cannabis inflorescences, leaves, stem barks, and roots for medicinal purposes. Sci. Rep. **10**, 3309. (10.1038/s41598-020-60172-6) 32094454 PMC7039888

[42] Gutiérrez A, Rodríguez IM, del Río JC. 2006 Chemical characterization of lignin and lipid fractions in industrial hemp bast fibers used for manufacturing high-quality paper pulps. J. Agric. Food Chem. **54**, 2138-2144. (10.1021/jf052935a) 16536588

[43] Peiretti PG. 2009 Influence of the growth stage of hemp (*Cannabis sativa* L.) on fatty acid content, chemical composition and gross energy. Agri. J. **4**, 27-31.

[44] Aizpurua-Olaizola O, Soydaner U, Oztürk E, Schibano D, Simsir Y, Navarro P, Etxebarria N, Usobiaga A. 2016 Evolution of the cannabinoid and terpene content during the growth of cannabis sativa plants from different chemotypes. J. Nat. Prod. **79**, 324-331. (10.1021/acs.jnatprod.5b00949) 26836472

[45] Nogueira AO, Oliveira YIS, Adjafre BL, de Moraes MEA, Aragao GF. 2019 Pharmacological effects of the isomeric mixture of alpha and beta amyrin from Protium heptaphyllum: a literature review. Fundam. Clin. Pharmacol. **33**, 4-12. (10.1111/fcp.12402) 30003594

[46] Chandra S, Lata H, Mehmedic Z, Khan IA, ElSohly MA. 2010 Assessment of cannabinoids content in micropropagated plants of Cannabis sativa and their comparison with conventionally propagated plants and mother plant during developmental stages of growth. Planta Med. **76**, 743-750. (10.1055/s-0029-1240628) 19950050

[47] Fadel D, Assaad N, Alghazal G, Hamouche Z, Lazari D. 2020 ‘Finola’ cannabis cultivation for cannabinoids production in Thessaloniki-Greece. J. Agri. Sci. **12**, 172. (10.5539/jas.v12n7p172)

[48] Glivar T, Erzen J, Kreft S, Zagozen M, Cerenak A, Ceh B, Tavcar Benkovic E. 2020 Cannabinoid content in industrial hemp (*Cannabis sativa* L.) varieties grown in Slovenia. Ind. Crops Prod. **145**, 112082. (10.1016/j.indcrop.2019.112082)

[49] Madaka F, Chankana N, Khamthong N, Maha A, Songsak T. 2021 Extraction and isolation of high quantities of cannabidiol, cannabinol, and deta-9-tetrahydrocannabinol from *Cannabis sativa* L. Malysian J. Anal. Sci. **25**, 867-881.

[50] Liu Y et al. 2022 Cannabis sativa bioactive compounds and their extraction, separation, purification, and identification technologies: an updated review. Trends Anal. Chem. 149, 116554. (10.1016/j.trac.2022.116554)

[51] Vuerich M, Ferfuia C, Zuliani F, Piani B, Sepulcri A, Baldini M. 2019 Yield and quality of essential oils in hemp varieties in different environments. Agronomy **9**, 356. (10.3390/agronomy9070356)

[52] Namdar D, Mazuz M, Ion A, Koltai H. 2018 Variation in the compositions of cannabinoid and terpenoids in *Cannabis sativa* derived from inflorescence position along the stem and extraction methods. Ind. Crops Prod. **113**, 376-382. (10.1016/j.indcrop.2018.01.060)

[53] Noppawan P, Bainier C, Lanot A, McQueen-Mason S, Supanchaiyamat N, Attard TM, Hunt AJ. 2022 Effect of harvest time on the compositional changes in essential oils, cannabinoids, and waxes of hemp (*Cannabis sativa* L.). *FigShare*. (10.6084/m9.figshare.c.6011489) PMC9198500 35719880

